# Supplementary figures and images for: Tet-dependent 5-hydroxymethyl-Cytosine modification of mRNA regulates axon guidance genes in Drosophila
Source: PLoS One. 2024 Feb 21;19(2):e0293894. doi: 10.1371/journal.pone.0293894 (PMC10881007; doi:10.1371/journal.pone.0293894)

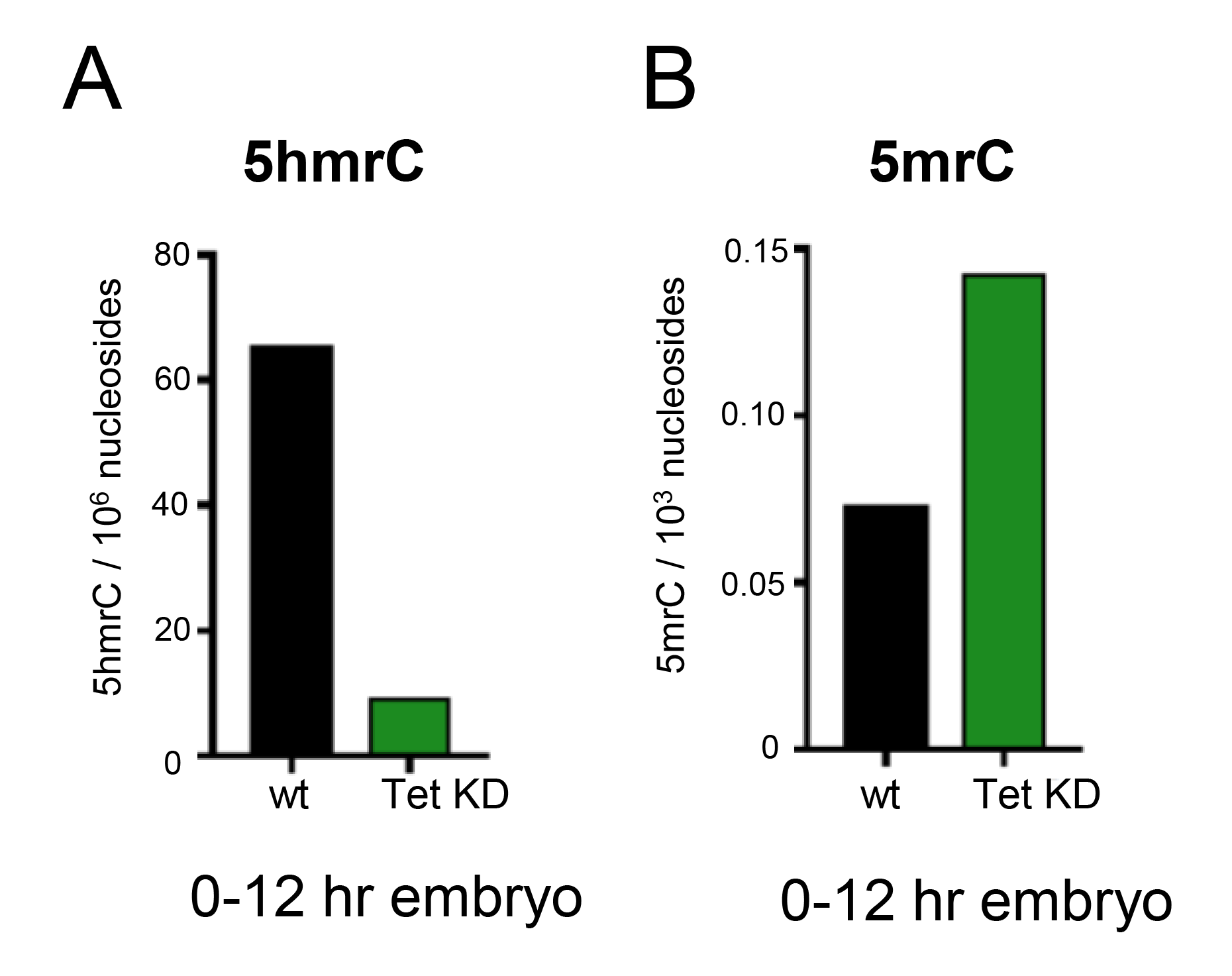

Supplement: S1 Fig — A. RNAi mediated KD of Tet alters the methylation status of Cytosine in total RNA by ultra-performance liquid chromatography tandem mass spectrometry. 5hmrC in total RNA isolated from wild-type and Tet KD embryos. B. 5mrC in total RNA isolated from wild-type and Tet KD embryos. (TIF) [file pone.0293894.s001.tif]

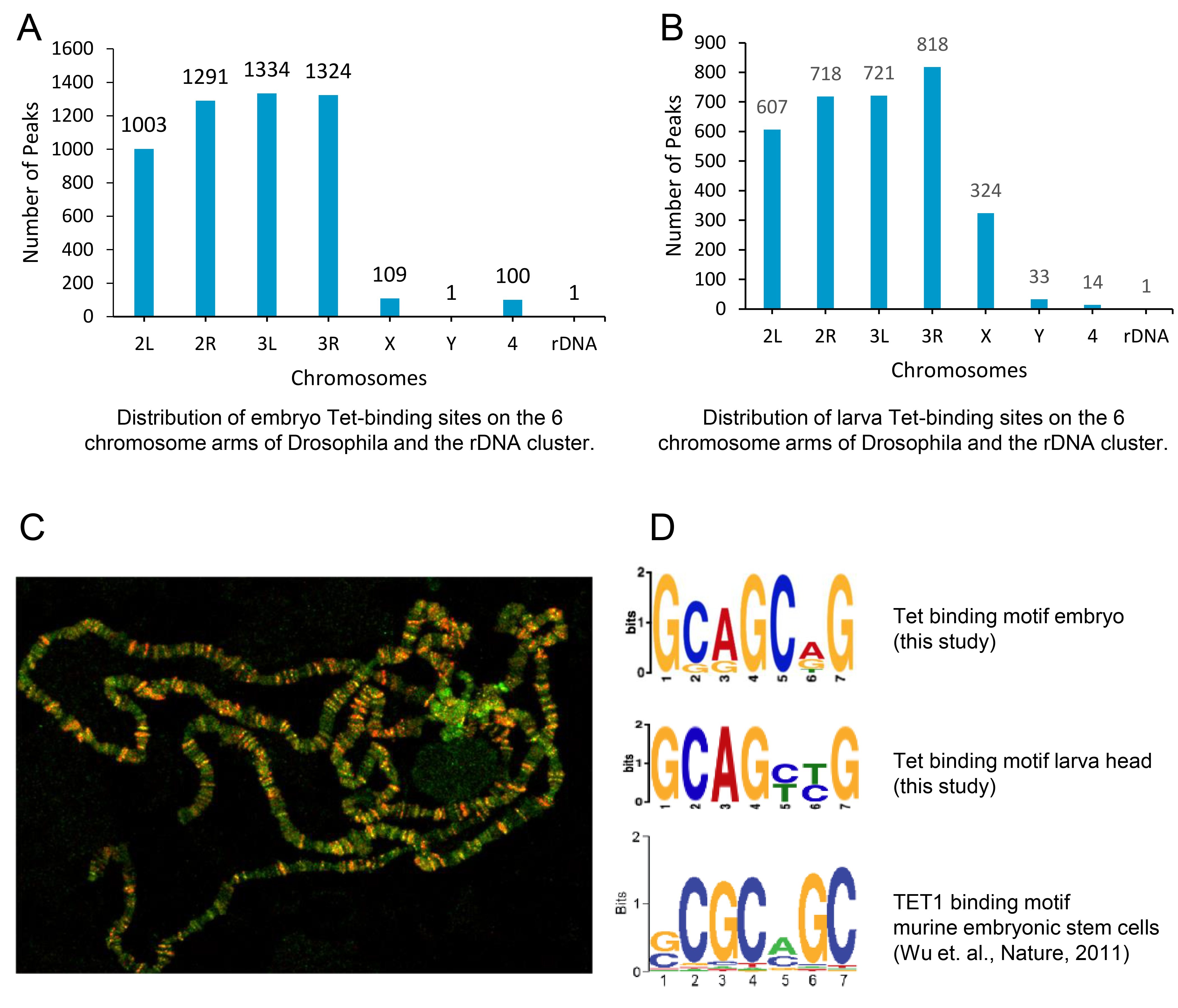

Supplement: S2 Fig — A. Distribution of Tet-binding sites in embryo DNA on the 6 chromosome arms of Drosophila and the rDNA cluster (177 peaks are found at the centromeres or ummapped_scaffolds); B. Distribution of Tet-binding sites in LBF DNA. on the 6 chromosome arms of Drosophila and the rDNA cluster (17 peaks are found at the centromeres or ummapped scaffolds); C. Localization of Tet CxxC DNA-binding domain (red) on polythene chromosome. Salivary gland chromosome from hsp70-GAL4::UAS-TetCxxC-RFP-Myc 3rd instar larvae were stained with anti-Myc (red, TetCxxC) and H3K4me3 (green). Control chromosomes from hsp70-GAL4 and UAS-TetCxxC alone show no Myc or RFP staining (not shown); D. Comparison of top DNA binding motives determined by Tet ChIP-seq in Drosophila 0–12 hr embryos, LBF, and murine ESCs. (TIF) [file pone.0293894.s002.tif]

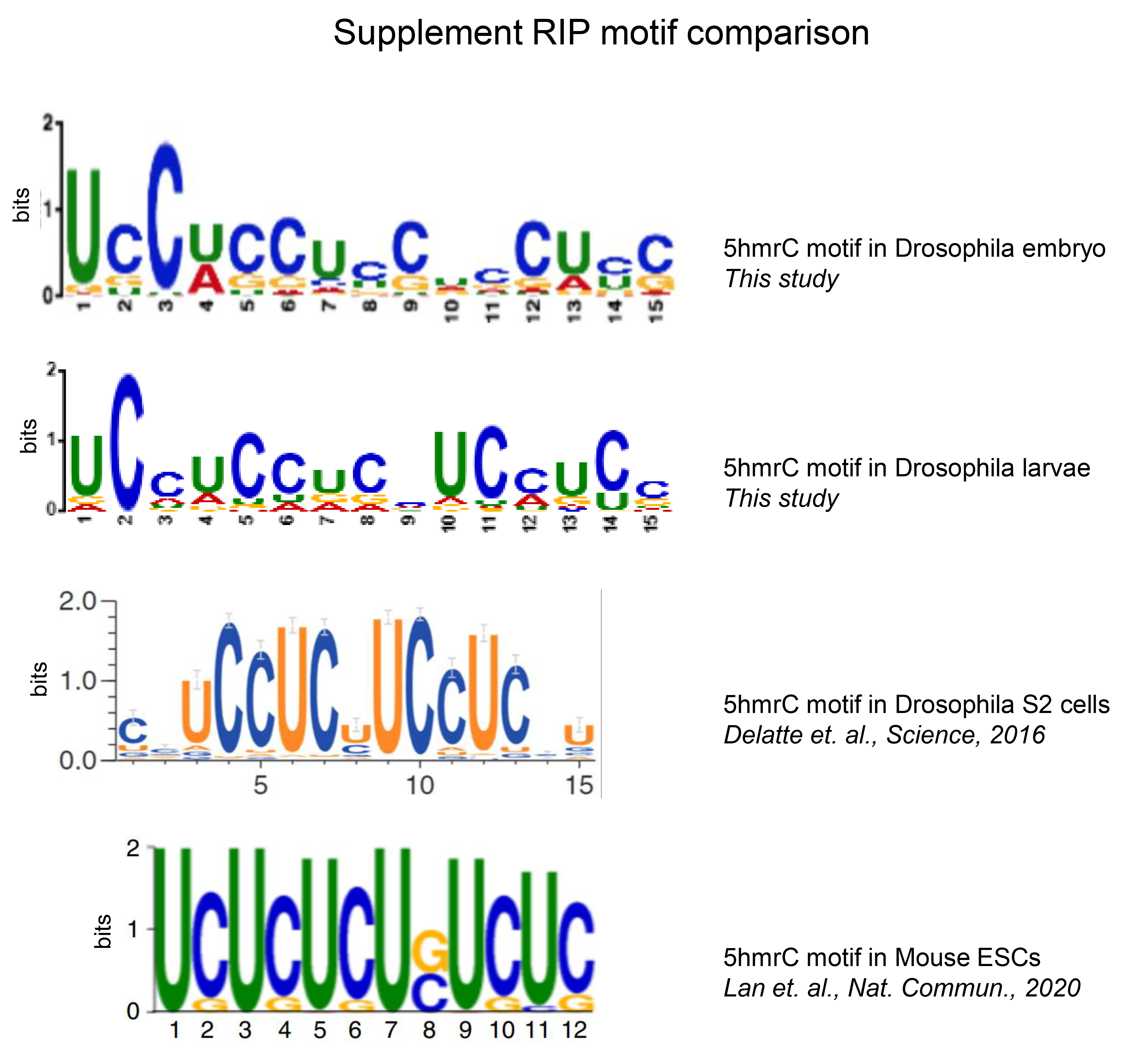

Supplement: S3 Fig — A. Comparison of the top sequence motives identified from 5hmrC peaks from Drosophila embryos. LBF, Drosophila S2 cells and mouse ESCs. (TIF) [file pone.0293894.s003.tif]

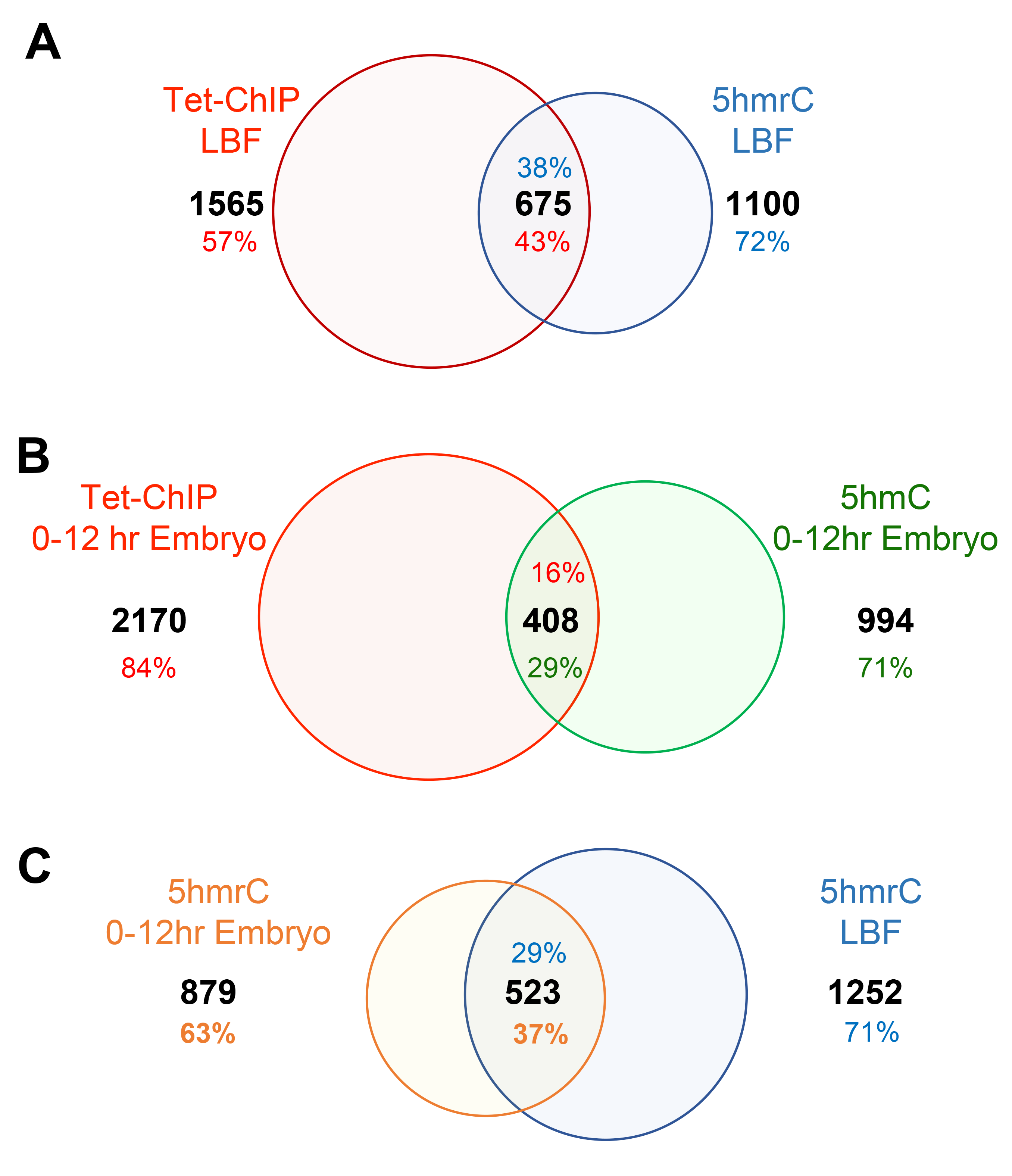

Supplement: S4 Fig — A., in LBF, B., in embryos. C. Overlap of 5hmrC modified transcripts in LBF and embryos. (TIF) [file pone.0293894.s004.tif]

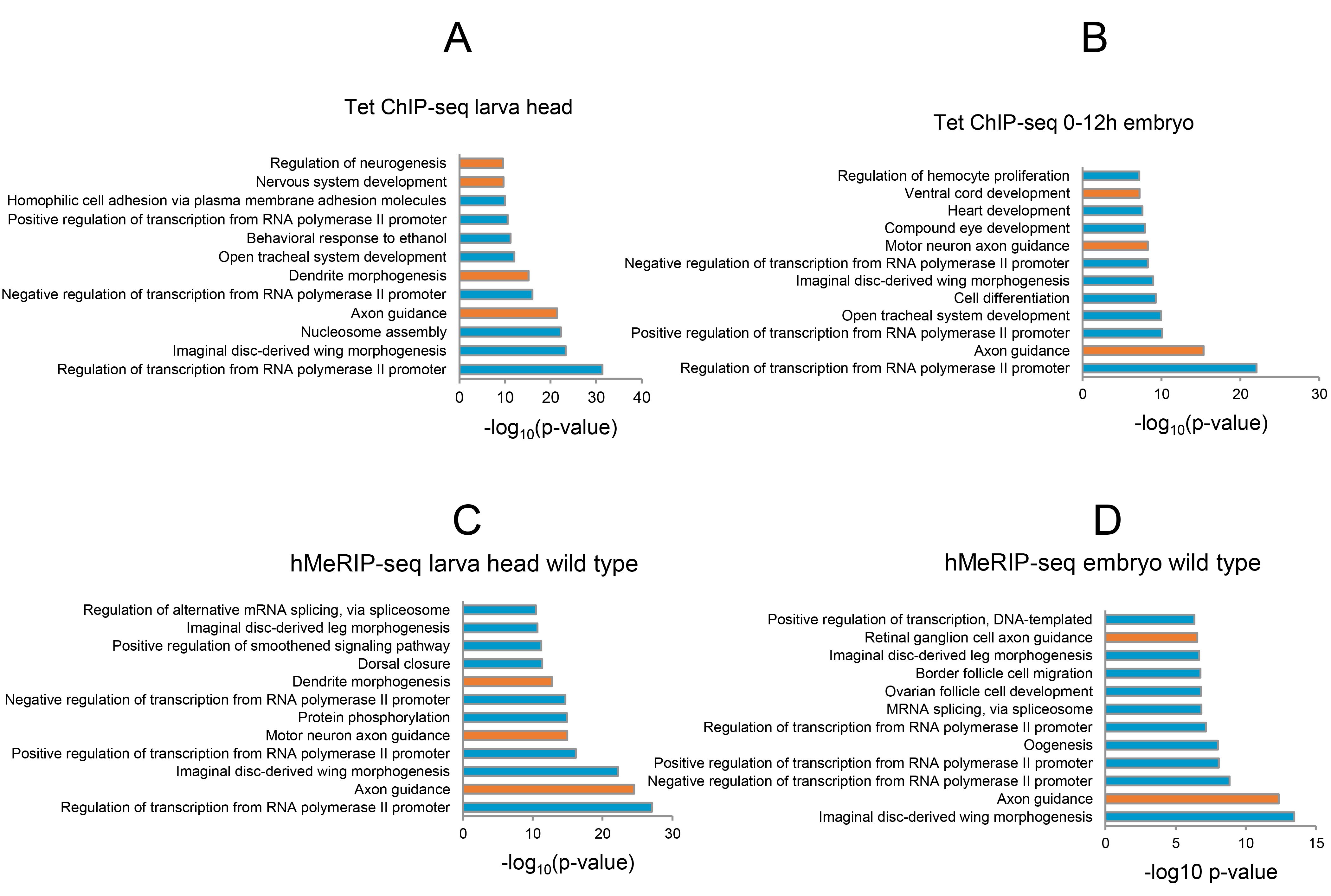

Supplement: S5 Fig — A. of genome-wide Tet peaks from LBF; B. of genome-wide Tet peaks from 0–12 hour embryos; C. of transcriptome-wide 5hmrC peaks in wild type LBF; D. transcriptome-wide 5hmrC peaks in Tetnull LBF. Note the consistency of the top two classes of genes. (TIF) [file pone.0293894.s005.tif]

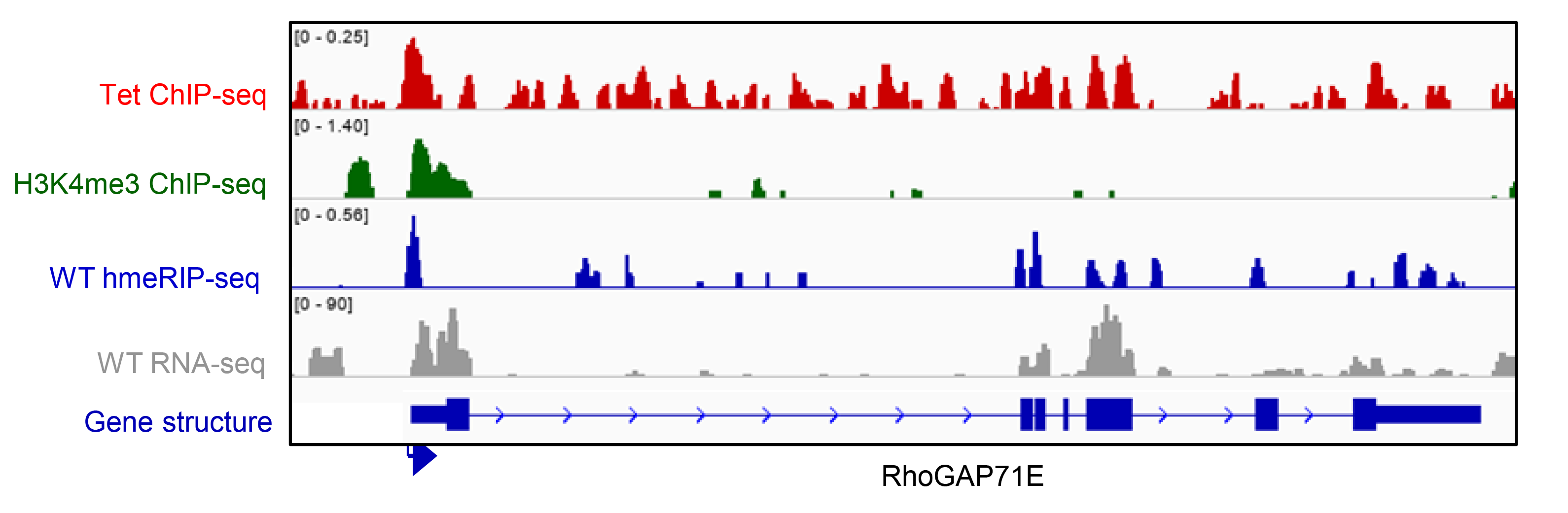

Supplement: S6 Fig — (TIF) [file pone.0293894.s006.tif]

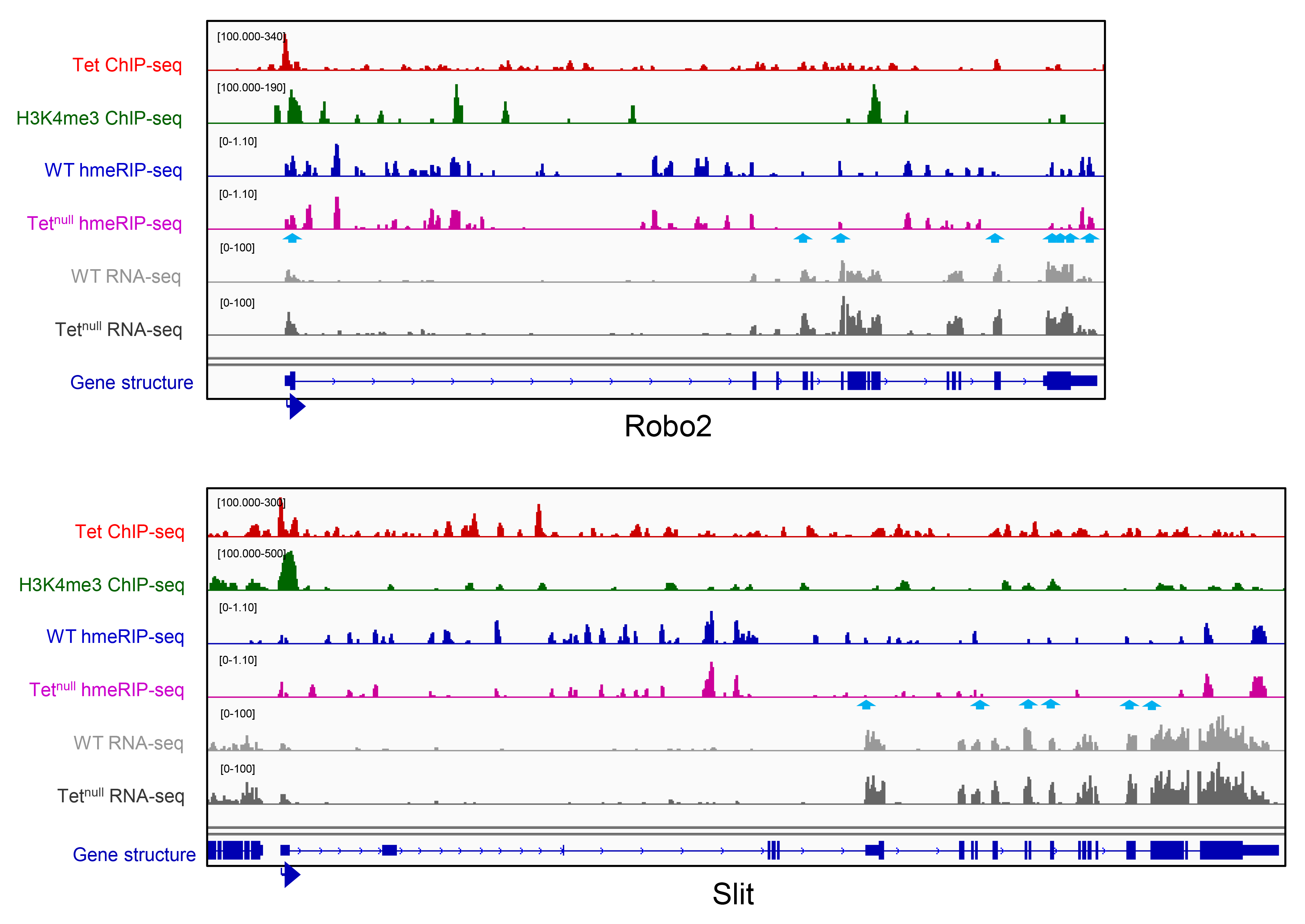

Supplement: S7 Fig — Cyan arrows show the reduction of 5hmrC peaks on exons. (TIF) [file pone.0293894.s007.tif]

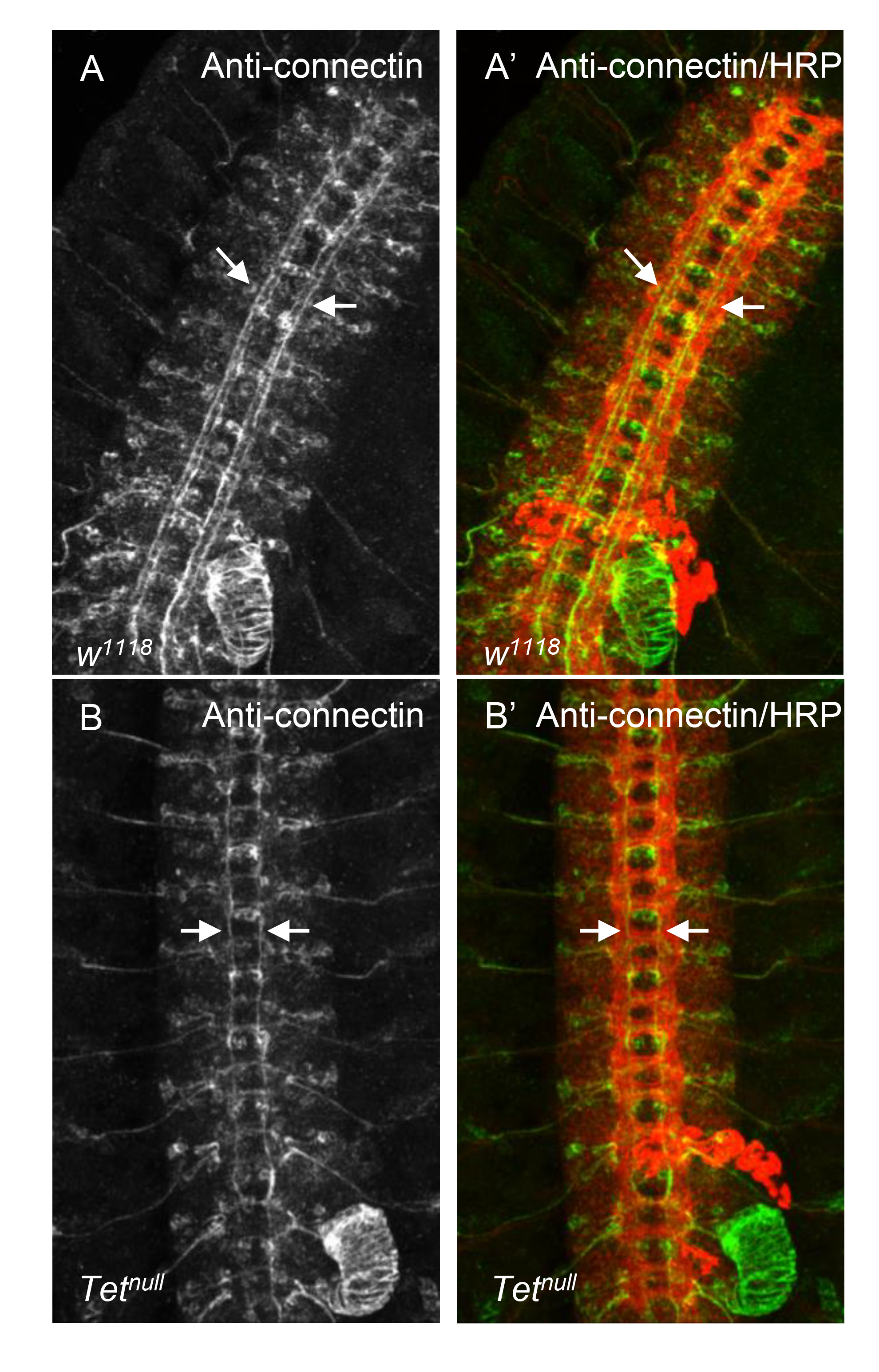

Supplement: S8 Fig — In wt (A) and Tetnull/Tetnull (B). A’,B’ show overlays of Connectin+ neurons (green) with the general neuronal marker HRP (red) in wt and Tetnull, respectively. Two Con+ tracks run within the longitudinal neuropil on each side of the midline of wt embryos (A, arrows) whereas Con+ neurons are present in only one medial track in Tetnull embryos (B, arrows). (TIF) [file pone.0293894.s008.tif]

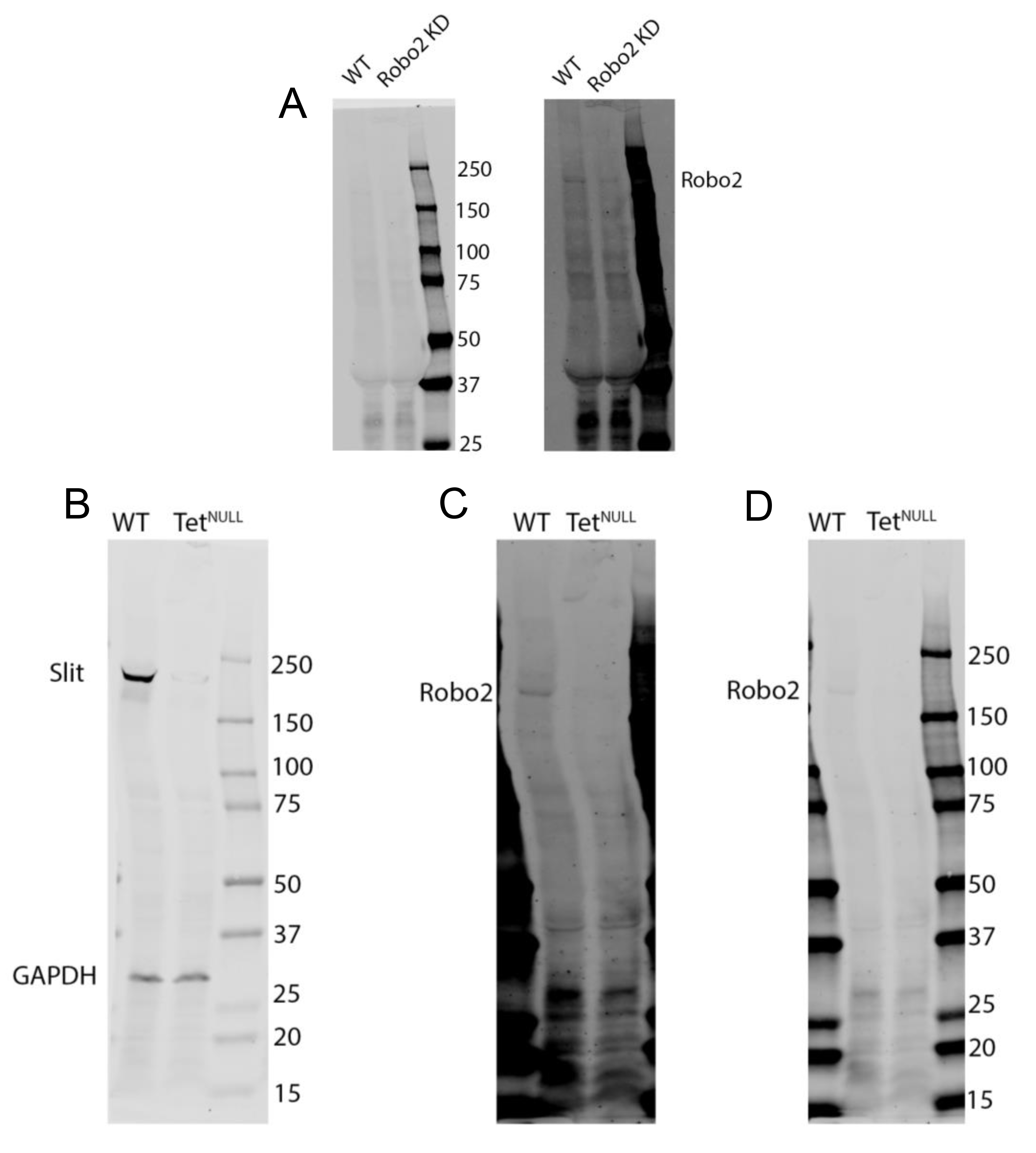

Supplement: S9 Fig — Full western blots: (A-B). To test the specificity of the anti-Robo2 antibody we collected 0–12 hour embryos of wt control and robo2 RNAi knockdown (KD). The wt lane shows a band between 150 and 250 kDa (comparing B to the same western blot in A with low intensity for ladder visualization) which is reduced in the robo2 KD lane indicating that is robo2 band and specificity of the Robo2 antibody. C. wt and Tetnull 3rd instar larval brain extracts probed with anti-Slit antibody. D. wt and Tetnull 3rd instar larval brain extracts probed with anti-Robo2 antibody. E. Same western blot in D. with low intensity for ladder visualization. (TIF) [file pone.0293894.s009.tif]

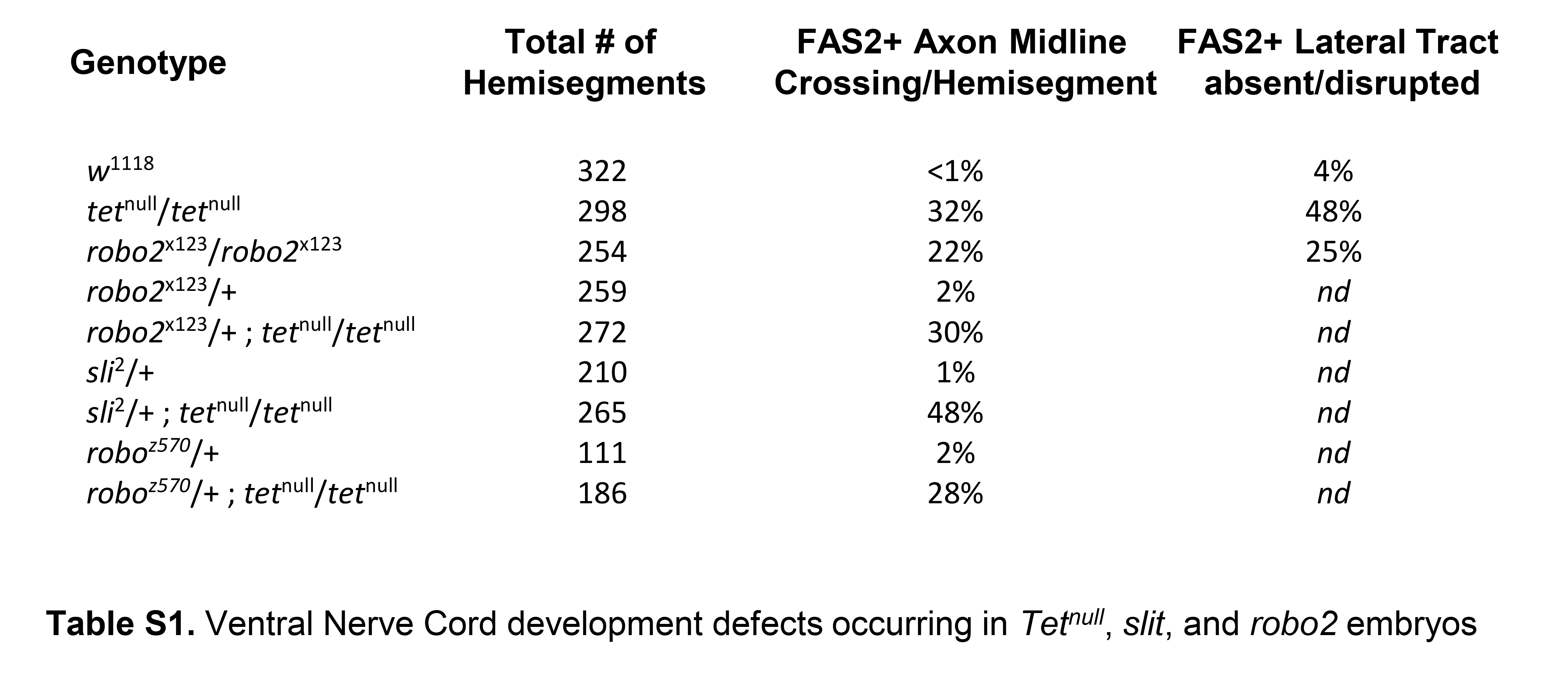

Supplement: S1 Table — Embryos prepared according to Materials and Methods were analyzed for midline crossing of Fas2+ neurons and the presence and integrity of the most lateral longitudinal Fas2+ and Connectin+ tracts. (TIF) [file pone.0293894.s010.tif]
